# Investigation of Newly Prepared Biodegradable ^32^P-chromic Phosphate-polylactide-co-glycolide Seeds and Their Therapeutic Response Evaluation for Glioma Brachytherapy

**DOI:** 10.1155/2018/2630480

**Published:** 2018-04-29

**Authors:** Guoqiang Shao, Yuebing Wang, Xianzhong Liu, Meili Zhao, Jinhua Song, Peiling Huang, Feng Wang, Zizheng Wang

**Affiliations:** ^1^Department of Nuclear Medicine, Nanjing First Hospital, Nanjing Medical University, Nanjing 210006, China; ^2^Department of Biochemistry, School of Medicine, Nankai University, Tianjin 300071, China; ^3^Department of Surgical Oncology, Bayi Hospital Affiliated to Nanjing University of Chinese Medicine, Nanjing 210006, China; ^4^Department of Invasive Technology, Nanjing First Hospital, Nanjing Medical University, Nanjing 210006, China; ^5^Department of Pathology, Medical College of Southeast University, Nanjing 210000, China

## Abstract

^32^P high-dose rate brachytherapy allows high-dose radiation delivery to target lesions with less damage to adjacent tissues. The early evaluation of its therapeutic effect on tumours is vital for the optimization of treatment regimes. The most commonly used ^32^P-CP colloid tends to leak with blind therapeutic area after intratumour injection. We prepared ^32^P-chromic phosphate-polylactide-co-glycolide (^32^P-CP-PLGA) seeds with biodegradable PLGA as a framework and investigated their characteristics* in vitro* and* in vivo*. We also evaluated the therapeutic effect of ^32^P-CP-PLGA brachytherapy for glioma with the integrin *α*v*β*3-targeted radiotracer ^68^Ga-3PRGD_2_. ^32^P-CP-PLGA seeds (seed group, SG, 185 MBq) and ^32^P-CP colloid (colloid group, CG, 18.5 MBq) were implanted or injected into human glioma xenografts in nude mice. Scanning electron microscopy (SEM) of the seeds, micro-SPECT imaging, and biodistribution studies were performed at different time points. The tumour volume was measured using a caliper, and ^68^Ga-3PRGD_2_ micro-PET-CT imaging was performed to evaluate the therapeutic effect after ^32^P intratumour administration. The delayed release of ^32^P-CP was observed with biodegradation of vehicle PLGA. Intratumoural effective half-life of ^32^P-CP in the SG (13.3 ± 0.3) d was longer than that in the CG (10.4 ± 0.3) d (*P* < 0.05), with liver appearance in the CG on SPECT. A radioactivity gradient developed inside the tumour in the SG, as confirmed by micro-SPECT and SEM. Tumour uptake of ^68^Ga-3PRGD_2_ displayed a significant increase on day 0.5 in the SG and decreased earlier (on day 2) than the volume reduction (on day 8). Thus, ^32^P-CP-PLGA seeds, controlling the release of entrapped ^32^P-CP particles, are promising for glioma brachytherapy, and ^68^Ga-3PRGD_2_ imaging shows potential for early response evaluation of ^32^P-CP-PLGA seeds brachytherapy.

## 1. Introduction

Glioblastoma multiforme (GBM) accounts for approximately 60% to 70% of malignant gliomas. The prognosis of patients with GBM remains dismal, with a median survival time of less than one year. This poor prognosis primarily reflects the high proliferative, infiltrative, and invasive properties of GBM [1]. Systemic administration of most potentially active drugs is not effective because of failing to cross the blood-brain barrier. Residual cells at the margins of the resection or GBM stem cells, a subpopulation of stem-like cells, frequently lead to tumour recurrence or metastasis.

Compared with external-beam radiation, brachytherapy has the advantage of delivering high doses of radiation to target sites without subjecting normal tissues to undue radiation and damage [2]. ^32^P is ideal because of its relatively long half-life of 14.28 day(s), maximum energy of 1.711 MeV, mean range of 3-4 mm in soft tissues, and high relative biological effect. Recent studies have focused on better vehicles or carriers, and many of these studies have used ^32^P bandages or films for the brachytherapy of superficial cancers and intracranial and spinal tumours [3–9]. However, the main hurdles to these vehicles were poor target ratios in the tumour, complex preparation, and serious side effects resulting from migration and permanent stagnation, such as lung migration and gastric fistula* in vivo* [7–9].

Polymers, such as poly(lactic acid) (PLA), poly(glycolic acid) (PGA), and their copolymer poly(lactide-co-glycolide) (PLGA), have extensively been used in drug delivery systems because of their biodegradability, tissue compatibility, and versatile degradation kinetics [10–13]. Compared with poly(L-lactide) (PLLA), PLGA has attracted more attention, reflecting its degradation rate and relative ease in manipulating its release of encapsulating drugs by changing the degradation-determining factors, such as its molecular weight, lactide/glycolide ratio, and degree of crystallinity [14, 15]. In a previous study, we completed the formulation and determined the fabricating parameters of ^32^P-CP-PLGA seeds (unpublished observations). As one potential biodegradable seed for brachytherapy, ^32^P-CP-PLGA is meaningful for further preclinical studies, and the therapeutic effect evaluation of ^32^P brachytherapy has rarely been reported.

Integrin *α*v*β*3, specifically targeted by the arginine-glycine-aspartic acid (RGD) tripeptide sequence, is highly expressed in both glioma cells and neovasculature but not in quiescent blood vessels. Integrin *α*v*β*3 targeted imaging had higher sensitivity in GBM than ^18^F-FDG, reflecting a clear lack of RGD affinity, particularly in brain areas where gliomas are prone to occur [16]. In an orthotopic U87MG human glioma mouse model, the tumour-to-brain ratio of radiolabelled RGD is approximately 20 times that of ^18^F-FDG [17]. ^68^Ga, easily accessible via elution from the ^68^Ge-^68^Ga generator, can produce Cerenkov radiation and allow the combination of PET-Cerenkov luminescence imaging (CLI) for both tumour diagnosis and visual guidance for further surgery [17]. ^68^Ga-labelled DOTA-3PRGD_2_ (^68^Ga-3PRGD_2_) was reported to reflect the tumour response to antiangiogenic therapy much earlier and more accurately than ^18^F-FDG PET imaging [18]. Integrin-targeted imaging detects early responses preceding clinical regression as early as day 3 after the initiation of abraxane treatment, while ^18^F-FDG imaging demonstrated increased tumour uptake on days 3 and 7 [19]. For ^32^P brachytherapy, early response is also important for treatment direction; however, few studies have been reported.

In our previous study, we found that radiolabelling RGD is excellent for monitoring *α*v*β*3 expression and glioma necrosis during tumour growth [20]. The present study reports (a) the preparation, biodegradation, and ultrastructural changes of ^32^P-CP-PLGA seeds and the biodistribution of ^32^P-CP particles both systemically and inside the tumour and (b) the anticancer effect on glioma in an animal model after intratumour seeds implantation and therapeutic effect evaluation with integrin *α*_v_*β*_3_ receptor targeting radiotracer ^68^Ga-3PRGD_2_.

## 2. Materials and Methods

### 2.1. Materials

^32^P-CP colloid, supplied by Beijing Atom High-Tech Co., Ltd., China, is a green colloid solution with radioactive chemical purity > 98%, particle size 20~50 nm, and specific radioactivity ~ 1850 MBq/ml. PLGA (50 : 50; MW-85,000 Da) was obtained from* Hefei University of Technology, China. *^68^Ge-^68^Ga generator (ITG, Germany), 3PRGD_2_, was donated by Dr. Shuang Liu (Purdue University). Nude mice (male, 14–16 g, 5-6 weeks) bearing human glioma (U87MG) xenografts in the flank were obtained from the Chinese Academy of Medical Sciences, China. All animal procedures were performed in accordance with the standards of the Institutional Animal Care and Utilization Committee. The following were used: hamster anti-integrin *β*_3_ antibody (1 : 100, BD Biosciences, San Jose, CA), rat anti-CD31 antibody (1 : 100, BD Biosciences), Cy3-conjugated goat anti-hamster secondary antibody (1 : 100, Jackson Immuno Research Inc., West Grove, PA), fluorescein- (FITC-) conjugated goat anti-rat secondary antibody (1 : 100, Jackson Immuno Research Inc., West Grove, PA), CRC-Enhanced *β* calculator (Capintec Corporation, USA), Gamma counter (TY6017, Wizard, PerkinElmer, USA), Electronic Balance (BS-110S, Sartorius Corporation, Germany), Scanning electron microscope (S-3000N, Hitachi High-Technologies Corporation, Japan), Olympic BX51 fluorescence microscope (Olympus America Inc., Center Valley, PA), micro-SPECT (Milabs, Utrecht, The Netherlands), and micro-PET/CT (Inveon, Siemens, Germany).

### 2.2. Preparation of ^32^P-CP-PLGA Seeds and* In Vitro* Study

A total of 100 mg of PLGA (0.1–1.0 *μ*m) and 2 ml of colloidal ^32^P-CP (1850 MBq/ml) were mixed with 2-3 ml of dehydrated alcohol as a dispersing agent. These components were blended and dried at 60°C in a vacuum drying oven for 9–12 h. Magnesium stearate (1.0–1.5 mg) served as a surface lubricant, and the ^32^P-CP-PLGA seeds were home-prepared using an automatic precise piercer. The physical characteristics of the ^32^P-CP-PLGA seeds were investigated.* In vitro* releasing experiments were performed by incubating the ^32^P-CP-PLGA seeds in 6 ml of PBS at 37°C for 90 day(s), and 0.2 ml of the sample was withdrawn every other day from the releasing solution after stirring to generate a homogenous mixture.

### 2.3. Radiopharmaceutical Administration and Biodistribution Study

Nude mice bearing U87MG human gliomas (tumour long diameter of 0.6–0.8 cm) were randomized into the ^32^P-CP-PLGA seed group (SG, *n* = 36) and the ^32^P-CP colloid group (CG, *n* = 36). One ^32^P-CP-PLGA seed (18.5 MBq) or 0.02 ml of colloidal ^32^P-CP (18.5 MBq) was implanted or slowly injected into the tumour core. The injection sites were gently compressed for 60 s. The mice were sacrificed at 0.5, 1, 2, 4, 8, 16, 32, 49, and 64 days (5 mice each time point each group) for biodistribution study and pathology examination.

Important organs were harvested, weighed, and counted for radioactivity using a *β* calculator or Gamma counter. Tumour xenografts harvested at 0.5, 1, 2, 4, 8, and 16 days were further divided into 3 parts according to their distances from the core of the tumour. The 3 parts of the tumour and adjacent tissues were weighed and counted for radioactivity. ^32^P distribution was expressed as the percent injected dose per gram (%ID/g). The remaining seeds and tumour tissues were prepared for SEM examination. For the mice sacrificed on day 64, micro-SPECT imaging was selectively performed, and faeces and urine were collected.

### 2.4. Micro-SPECT and Micro-SPECT/CT Imaging

Anesthesia of mice (SG and CG) was induced using isoflurane. Micro-SPECT images were obtained on days 1, 4, 16, and 64 using a micro-SPECT scanner equipped with a 0.6 mm multipinhole collimator. The specified ^32^P-CP-PLGA seeds and ^32^P-CP colloids, same as those used in the SG and CG, were served as markers and imaged, respectively. Regions of interest (ROIs) of the targeted tumour (T) and the marker (T_mk_) were drawn to estimate the T/T_mk_ ratio and intratumoural effective half-life of ^32^P-CP. After SPECT acquisition (75 projections over 30 min per frame, 2 frames), SPECT reconstruction was performed using a POSEM (pixelated ordered subsets by expectation maximization) algorithm with 6 iterations and 16 subsets. Micro-CT imaging was added on day 1 using the “normal” acquisition settings at 45 kV and 500 A. CT data were reconstructed using a cone-beam filtered back-projection algorithm (NRecon v1.6.3, Skyscan). After reconstruction, the SPECT and CT data were automatically coregistered according to the movement of the robotic stage and then resampled to equivalent voxel sizes. Coregistered images were further rendered and visualized using PMOD software (PMOD Technologies, Zurich, Switzerland).

### 2.5. Therapeutic Effect of ^32^P-CP-PLGA Seeds for Gliomas

39 nude mice bearing U87MG gliomas (long diameter of 0.6–0.8 cm) were randomly divided into ^32^P-CP-PLGA seed group (18.5 MBq, *n* = 17), ^32^P-CP colloid group (18.5 MBq, 0.02 ml, *n* = 5), and P-CP-PLGA seed group (no radioactivity, *n* = 17). Tumour diameter was measured with digital caliper every other day and tumour volume was estimated by the formula (length × width^2^)/2 (5 mice each group for three group). ^68^Ga-3PRGD_2_ micro-PET-CT imaging was performed on days 0.5, 2, 8, and 16 after seeds administration (*n* = 5 of each group). Four mice were sacrificed on days 0.5, 2, and 4 in SG and P-CP-PLGA seed group and tumour xenografts were harvested for immunohistochemistry (IHC) examination.

### 2.6. ^68^Ga-3PRGD_2_ Micro-PET-CT Imaging

We prepared ^68^Ga-3PRGD_2_ as previously described [18]. In brief, ^68^GaCl_3_ was eluted from ^68^Ge/^68^Ga-generator with 0.05 M HCl. The conjugation of 3PRGD_2_ with DOTA-OSu was performed for the synthesis of DOTA-3PRGD_2_. The DOTA-3PRGD_2_ and ^68^GaCl_3_ were mixed and heated in a water bath at 90°C for 15 min. The radiochemical purity was then determined with radio-HPLC.

0.1 mL of ^68^Ga-3PRGD_2_ (74 MBq/mL) was injected via tail vein under isoflurane anesthesia. Ten-minute static PET scans were acquired and images were reconstructed by an OSEM 3D (Three-Dimensional Ordered Subsets Expectation Maximum) algorithm followed by MAP (Maximization/Maximum A Posteriori). The 3D ROIs were drawn over the tumour guided by CT images and tracer uptake was measured using the software of Inveon Research Workplace (IRW) 3.0. Mean standardized uptake values (SUV) were determined by dividing the relevant ROI concentration by the ratio of the injected activity to the body weight.

### 2.7. Seeds Ultrastructure Changes and Glioma Integrin *α*v*β*3 Expression

SEM examination was performed to investigate the ultrastructure changes of ^32^P-CP-PLGA seeds. Frozen glioma tissues were cut into 5 *μ*m slices and blocked with 10% goat serum for 30 min and then incubated with the hamster anti-integrin *β*_3_ antibody and rat anti-CD31 antibody for 1 h at room temperature. After incubating with the Cy3-conjugated goat anti-hamster and fluorescein- (FITC-) conjugated goat anti-rat secondary antibodies and washing with PBS, the fluorescence was visualized using a fluorescence microscope.

### 2.8. Statistical Analysis

Data are shown as mean ± SD. Differences in tissues uptake between SG and CG were statistically analyzed at each time point with SPSS 11.5 software. Student's* t-test *was used for statistical analysis. *P values *less than 0.05 were considered statistically significant.

## 3. Results

### 3.1. Basic Characteristics and Biodegradation of ^32^P-CP-PLGA Seeds

**Figure 1:**
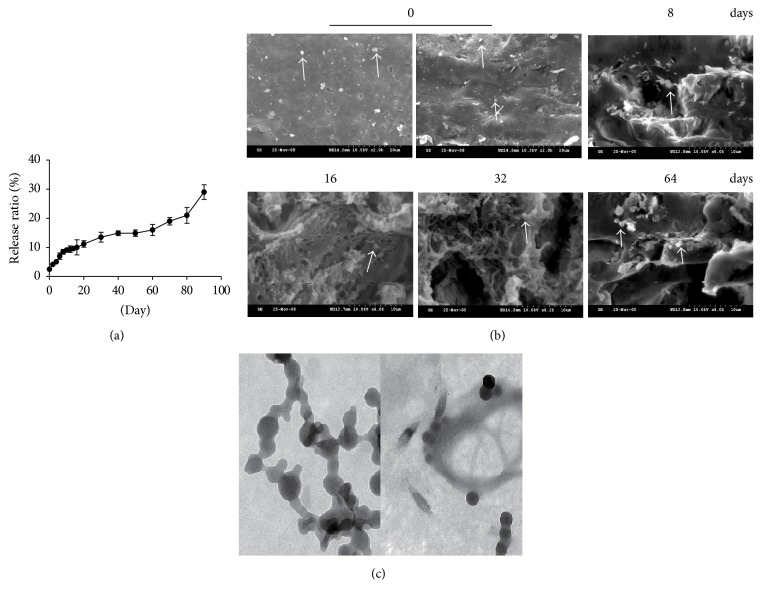
Time-release curve of ^32^P-CP particles from ^32^P-CP-PLGA seeds and ultrastructure changes of ^32^P-CP-PLGA seeds by SEM* in vitro*. (a)* In vitro* releasing experiment was performed by incubating the ^32^P-CP-PLGA seeds in 6.0 ml of PBS at 37°C for 90 day(s). (b) SEM was performed before day 0 (left: surface; right: cross-section) and 8, 16, 32, and 64 day(s) after intratumoural implantation. ^32^P-CP particles, indicated with white arrow, encapsulated in the seeds and released with the structure erosion of carrier PLGA polymer. (c) The ultrastructure of ^32^P-CP particles released from the PLGA* in vitro* (50 nm).

^32^P-CP-PLGA seeds are cylindrical with a diameter of (0.9 ± 0.02) mm, length of (1.9 ± 0.2) mm, weight of (1.2 ± 0.15) mg each, hardness of (5.7 ± 1.4) N, and apparent radioactivity of (18.5 ± 3.7) MBq. The release of ^32^P-CP-PLGA seeds can be divided into the rapid release phase (0~8 d, phase I), the stable release phase (8~60 d, phase II), and the disintegration release phase (60 d ~, phase III) according to time-release curve slope (Figure 1(a)).

Figure 1(b) displays the SEM results of ^32^P-CP-PLGA seeds before and 8, 16, 32, and 64 days after intratumour implantation. The seed was uniform and dense in texture, showing a mosaic of ^32^P-CP particles prior to implantation. An increasing number of micropores, tunnels, cellular structures, cavities, and fragments were observed with increasing seed brittleness and inability to maintain shape during release.  Figure 1(c) displays the ultrastructure of ^32^P-CP particles before the preparation of ^32^P-CP-PLGA seeds and those released from the ^32^P-CP-PLGA seeds.

### 3.2. Biodistribution of ^32^P-CP Particles

**Figure 2:**
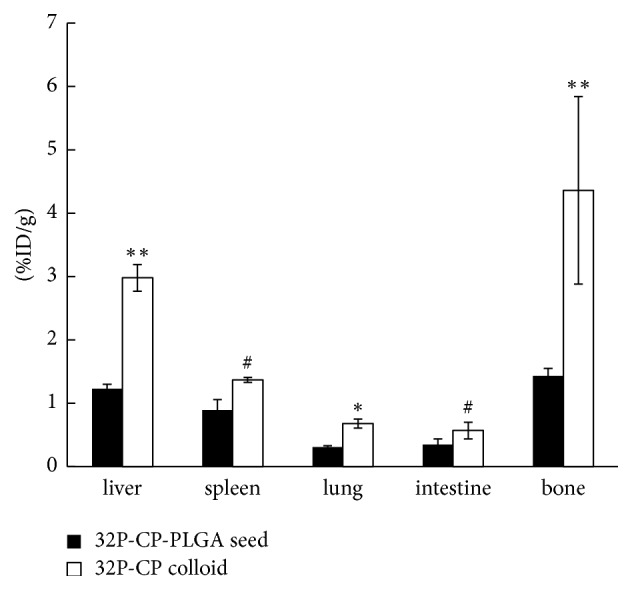
Bar graphs of the peak uptake of ^32^P-CP in important organs after intratumour administration of ^32^P-CP-PLGA seeds and ^32^P-CP colloids (*∗∗* indicates *P* < 0.01, *∗* indicates *P* < 0.05, and # indicates *P* > 0.05).

The biodistribution of ^32^P-CP in SG and CG is shown in Tables 1 and 2. The released radioactivity reached up to (7.3 ± 1.4)% on day 8 and increased to approximately (21 ± 3.8)% on day 64, among which approximately (14.6 ± 2.7)% remained in the tumour, (5.2 ± 1.3)% was primarily aggregated in the liver, spleen, and bone, and approximately (3.1 ± 0.86)% was eliminated in faeces and urine. Leakage of ^32^P-CP colloid was detected around the tumour xenograft in the CG. Compared with the CG, the peak uptake of ^32^P-CP in the liver (3.1 ± 0.3)%, spleen (1.3 ± 0.1)%, and bone (4.3 ± 1.8)% was significantly less in the SG [(1.2 ± 0.1)% in liver, (0.8 ± 0.2)% in spleen, and (1.5 ± 0.1)% in the bone, all of *P* < 0.05] (Figure 2). Intratumoural radioactive effective half-life of ^32^P-CP in the SG (13.3 ± 0.3 d) was longer than that in the CG [(11.4 ± 0.7) d, *P* < 0.05]. Pathological examination of the liver, spleen, lung, and bone demonstrated no abnormality in the SG.

### 3.3. Radioactivity Gradient Developed in Tumour and Surrounding Tissues

**Figure 3:**
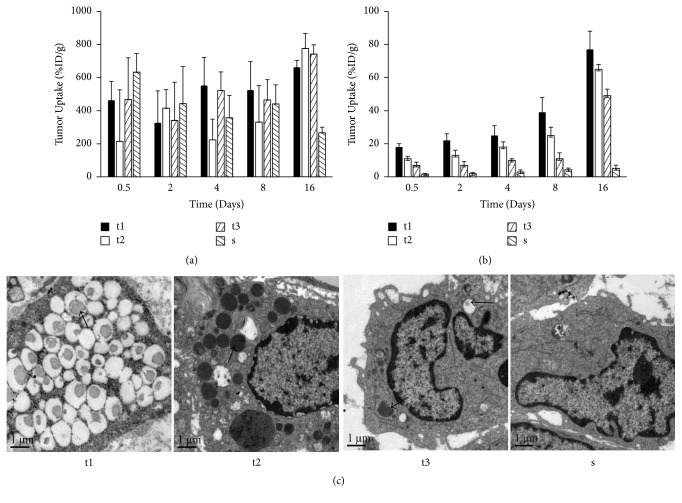
Bar graphs and SEM results of ^32^P-CP distribution in tumour and adjacent normal tissues at different time points after the intratumoural administration of SG and CG. (a) ^32^P-CP distribution in tumour and adjacent normal tissues at different time points in CG. The regional peak uptake in tumour tissues (t2) on day 16 was higher than day 8 (t1) (# means *P* < 0.05) [t1, t2, and t3 indicate tumour tissues at different distance from the seed (SG) or the core of the tumour (CG), near to far, and “s” indicates tissues surrounding the tumour]. (b) ^32^P-CP distribution in tumour and adjacent normal tissues in the SG (significant difference between ti and t2, t2 and t3, and t3 and s, *P* < 0.05) (c). SEM results of tumour and adjacent normal tissues (t1, t2, t3, and s). The ^32^P-CP particles are indicated with a black arrow. Irregularity of radioactivity distribution can be observed in the tumour and adjacent tissues in the CG, while a radioactivity gradient formed in the SG. The results were confirmed by SEM.

Figure 3 demonstrated the distribution of the released ^32^P-CP particles in tumour and adjacent normal tissues in the CG or SG after eradication and radioactivity counting of the remaining ^32^P-CP-PLGA seeds. Most of released ^32^P-CP particles in the SG were stagnant in the tumour core with a decreasing gradient towards the periphery in the SG (Figure 3(b)). This finding was confirmed by using SEM (Figure 3(c)). The gradient existed for approximately 2 weeks and became insignificant on day 14. The %ID/g tumour uptake of ^32^P-CP significantly increased on day 16, primarily reflecting tumour shrinkage. Conversely, the intratumour distribution of ^32^P-CP in the CG was irregular and a slight increase of %ID/g tumour uptake was observed with tumour shrinkage. The regional peak uptake in tumour tissues (t2) on day 16 was higher than day 8 (t1) (Figure 3(a)).

**Figure 4:**
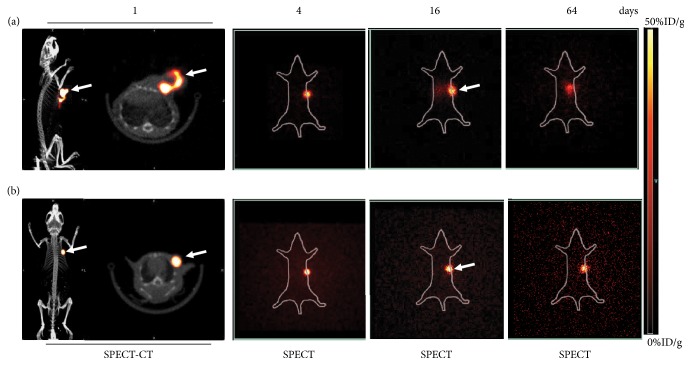
Representative micro-SPECT/CT or micro-SPECT images in the SG and CG. Micro-SPECT/CT images on day 1 and micro-SPECT images on days 4, 16, and 64 were obtained after intratumoural injection of colloidal ^32^P-CP ((a) ^32^P-CP colloids were in irregular shapes with leak from injection site and much notable abnormal distribution and liver appearance) and intratumour implantation of ^32^P-CP-PLGA seeds ((b) ^32^P-CP-PLGA seeds demonstrated the shape of a dot. No significant difference or appearance of other organs was observed until 64 days). The white arrows refer to tumors.

*Micro-SPECT/CT Imaging*. ^32^P bremsstrahlung facilitates SPECT-CT imaging to monitor the intratumour administration of ^32^P-CP colloid (Figure 4(a)) and ^32^P-CP-PLGA seeds (Figure 4(b)). As shown in Figure 4(b), ^32^P-CP-PLGA seeds showed dot-like focal radioactivity accumulation on days 1 and 4. The released ^32^P-CP showed a vague appearance of tumour on days 16 and 64 in the SG. ^32^P-CP colloids displayed irregular shapes in and/or close to the tumour location, with abnormal distribution in the liver in the CG on day 16, and this effect was even more apparent on day 64. The T/T_mk_ decreased to 89.3% ± 2.4% (SG) and 65.8% ± 3.1% (CG) on day 64 based on micro-SPECT/CT images.

### 3.4. ^68^Ga-3PRGD_2_ Micro-PET/CT Imaging and Tumour Growth Monitoring

**Figure 5:**
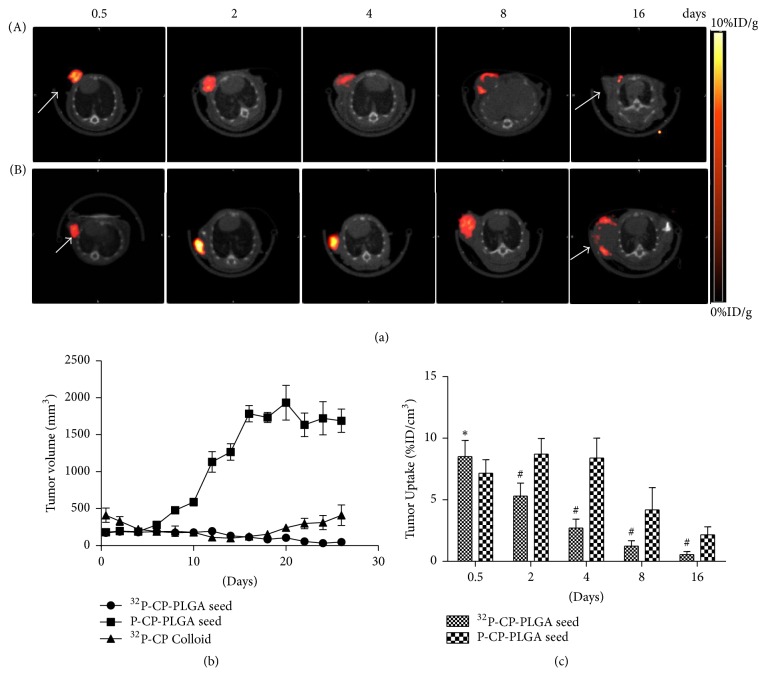
^68^Ga-3PRGD_2_ micro-PET-CT imaging during ^32^P brachytherapy and tumour volume measurement using a caliper. (a) The ^68^Ga-3PRGD_2_ micro-PET/CT transverse images of nude mice bearing glioma on days 0.5, 2, 4, 8, and 16 after intratumoural implantation of radioactive ^32^P-CP-PLGA seeds (A) and nonradioactive P-CP-PLGA seeds (B). (b) Tumour volume was measured using a caliper every other day after intratumoural administration of ^32^P-CP-PLGA seeds, ^32^P-CP colloid, and nonradioactive P-CP-PLGA seed. (c) Tumour uptake of ^68^Ga-3PRGD_2_ was quantified based on micro-PET/CT imaging and compared between radioactive and nonradioactive seeds group. Tumour uptake (%ID/g) of ^68^Ga-3PRGD_2_ was significantly different at the same points. In ^32^P-CP-PLGA seeds group, distribution heterogeneity of ^68^Ga-3PRGD_2_ was observed on day 2 and was even significant and toroidal on day 4, with slight accumulation of radiotracer on day 8 and being almost negative on day 16. The distribution of ^68^Ga-3PRGD_2_ was uniform at 8 days after nonradioactive seed implantation. Internal absence was observed on day 16, reflecting ulcer formation. # means tumour uptake of ^68^Ga-3PRGD_2_ in P-CP-PLGA seeds group was significantly lower than ^32^P-CP-PLGA seeds group (*P* < 0.05) while *∗* means tumour uptake of ^68^Ga-3PRGD_2_ in P-CP-PLGA seeds group was significantly higher than ^32^P-CP-PLGA seeds group (*P* < 0.05). The white arrows refer to tumors.

As shown in Figure 5, the tumour uptake heterogeneity of ^68^Ga-3PRGD_2_ was observed on days 2 and 4 with internal absence on day 8. Scar formation was observed on day 16 in the SG. In the P-CP-PLGA seed group, the distribution of ^68^Ga-3PRGD_2_ was uniform for 8 day(s), and internal heterogeneity was observed on day 8, reflecting natural necrosis. The tumour uptake (%ID/cm^3^) of ^68^Ga-3PRGD_2_ in the SG (8.50 ± 1.32) was higher than that in the P-CP-PLGA group (7.15 ± 1.49, *p* < 0.05) on day 0.5, while the tumour uptake in the SG (5.13 ± 1.06) was lower than that in the P-CP-PLGA group (8.16 ± 1.26, *p* < 0.01) on day 2. The tumour time-growth curve displayed no significant changes during 10 days in the SG or CG after ^32^P administration, and 40% (2/5) of the mice demonstrated tumour growth near the scar on day 20.

### 3.5. Immunohistochemistry Examination

**Figure 6:**
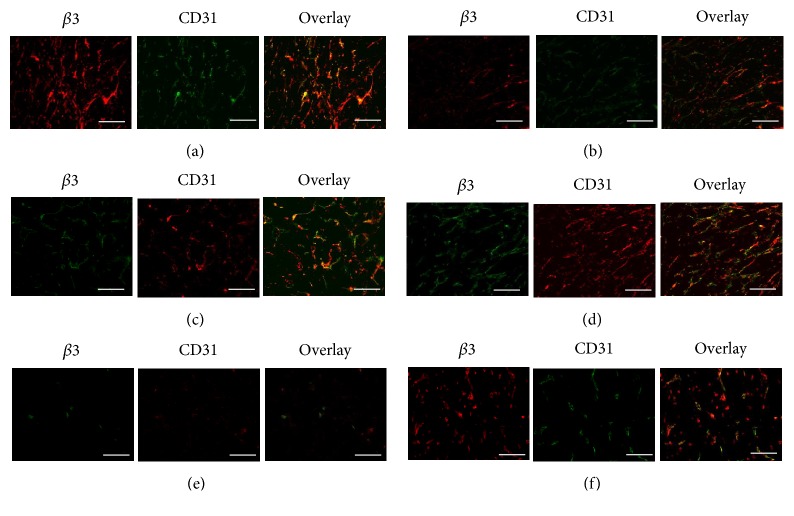
Representative immunohistochemical images for frozen glioma slices from xenografted glioma tumours at different time points (day 0.5: (a, b); day 2: (c, d); day 4: (e, f)) after intratumoural implantation of radioactive ^32^P-CP-PLGA seeds (a, c, e) and nonradioactive P-CP-PLGA seeds (b, d, f). The CD31 was used to label the tumour endothelial cells on blood vessels. The integrin *β*3 was visualized with Cy3 (red), and CD31 was visualized with fluorescein isothiocyanate (green) under an Olympus fluorescence system, scale bar: 50 *μ*m.

Immunofluorescence staining was performed to examine the *α*_v_*β*_3_ and CD31 expression levels in tumour tissues from the xenografted gliomas in the SG and P-CP-PLGA group on days 0.5, 2, and 4; as shown in Figure 6, compared with the P-CP-PLGA seed group, the quantitation of the glioma anti-*α*v*β*3 immunofluorescence levels in the SG revealed a 1.37-fold (±0.15) mean increase (±SD) on day 0.5 and 0.74-fold (±0.22) and 0.15-fold (±0.05) decreases on days 2 and 4, respectively. IHC examination was not performed on days 8 and 16 due to increased areas of necrosis and scar formation.

## 4. Discussion

^32^P-CP-PLGA seed is a biodegradable dosage form of ^32^P, utilizing PLGA as the controlled release carrier for drug delivery. In addition to its easy preparation in large amounts, the main advantage of ^32^P-CP-PLGA seeds is their delivery of high-dose radiation to the tumour and long intratumoural radioactive effective half-life with minimal distribution and damage to systemic tissues. A radioactivity gradient formed inside the tumour with potential effect to enhance the intratumour fire effect, reduce damage to adjacent tissues, and avoid the implantation of low radioactivity seeds in the peripheral part of the tumours.

No significant difference was observed in tumour uptake of ^32^P-CP particles in the SG and CG immediately after administration, except for the occurrence of leakage of colloidal ^32^P-CP in the CG. However, the dynamic radioactivity distribution indicated more retention and longer effective half-life of ^32^P-CP in the tumour and less or even undetectable distribution in other tissues in the SG compared with that in the CG. The principal mechanism likely involved the arrest of ^32^P-CP particles inside the seeds using the framework of PLGA, and their release and slow drainage was determined by the biodegradation of PLGA. A radioactivity concentration gradient, formed by the ^32^P-CP particles released from the ^32^P-CP-PLGA seeds, was observed inside the tumour. The tumour uptake (%ID/g) was extremely high, primarily because more than 95% (%ID) of the implanted ^32^P remained in the small tumour tissues (approximately 0.2 cm^3^) during the first few days. The increased uptake primarily reflected tumour shrinkage on day 16 and scar formation. The released ^32^P-CP particles increase the killing range and enhance the therapeutic effect without noticeable damage to adjacent tissues. The gradient gap was predominant in the first few days and narrowed with increasing release of ^32^P-CP particles and tumour shrinkage. However, the irregular and uneven intratumour distribution of ^32^P-CP colloids in the CG led to a therapeutic blind area, tumour relapse, and leakage from the injection site, resulting in damage to the adjacent tissues.

The ^32^P-CP particles drained from the tumour were primarily distributed throughout reticuloendothelial systems, such as the liver in the CG, and were less than reported [9]. This effect potentially reflects the use of gelatine sponge to prevent or reduce the leakage of ^32^P-CP colloids. The uptake of ^32^P-CP particles in normal tissues peaked at 16 days and likely involved the following three mechanisms. First, ^32^P-CP particles at the surface or inlaying the outer 1/3 of the seeds tended to be released, while those inlaid near the core stagnated inside, although there was gradual formation of tunnels and cavities. Second, the decrease or lack of regional circulation around the seeds resulting from tumour tissue necrosis and scar formation may inhibit the drainage of released ^32^P-CP particles. Third, the experimental period used in the present study may not be long enough. Pathological changes of important organs were absent in the SG.

^32^P bremsstrahlung SPECT imaging facilitates the positioning, effective for half-life evaluation under physical conditions [21]. Any immigration or abnormal uptake can easily be detected, and SPECT-CT fusion techniques with a certain postprocessing attenuation correction algorithm may contribute to precise localization, dosiology studies, and future establishment of brachytherapy in clinical settings [6].

^18^F-FDG PET-CT imaging was widely used to monitor therapeutic effect of tumour treatment. However, two potential disadvantages limit its use in gliomas. One disadvantage is the high distribution of glioma in normal brain tissues, particularly in the cerebral cortex, basal ganglia, and thalamus, where glioma is prone to occur. The other disadvantage is the increased accumulation of tumour tissue when inflammatory cells, such as macrophages, are present, particularly at the early stages of treatment [19]. ^18^F-FPPRGD_2_ is superior to ^18^F-FDG because the uptake of ^18^F-FPPRGD_2_ is not influenced by tumour-associated macrophages after chemotherapy, and the remarkable distribution of ^68^Ga-3PRGD_2_ in the U87MG xenografts is observed with low background in the normal brain tissues compared with ^18^F-FDG [17, 19]. ^68^Ga is easily accessible from a ^68^Ge-^68^Ga generator and showed similar labelling characteristics with therapeutic isotopes, such as ^177^Lu, with DOTA and NOTA as linkers. In the present study, the initial increased uptake of ^68^Ga-3PRGD_2_ in glioma was observed on day 0.5 after ^32^P-CP-PLGA seed implantation. The possible mechanism involves the rapid upregulation of integrin expression induced by a low-to-intermediate dose of radiation, as previously reported [22]. Thus, the increased glioma uptake of ^68^Ga-3PRGD_2_ on day 0.5 in the SG is possibly due to radiation, while the decrease on day 2 is due to cell damage resulting from accumulated ^32^P radiation and therapeutic effects [23]. This effect occurred much earlier than the tumour volume reduction and was confirmed using IHC. ^68^Ga-3PRGD_2_ imaging has potential to serve as an important tool for the therapeutic evaluation of ^32^P brachytherapy of glioma as early as day 2. Thus, the precise relationship between the *β*^−^ radiation dose and integrin expression upregulation needs further study.

## 5. Conclusions

^32^P-CP-PLGA seeds, controlling the release of entrapped ^32^P-CP particles, are promising for glioma brachytherapy. ^68^Ga-3PRGD_2_ imaging shows potential for early response evaluation of ^32^P-CP-PLGA seeds brachytherapy.

## Figures and Tables

**Table 1:** Biodistribution of ^32^P-CP particle in nude mice bearing U87MG glioma after intratumor implantation of ^32^P-CP-PLGA seed (*n* = 5).

Tissue	Radioactivity distribution (%ID/g)									
		0.5 d	1 d	2 d	4 d	8 d	16 d	32 d	48 d	64 d
Tumor	Mean 1 SD	556.686 24.016	547.778 61.301	626.172 76.528	666.063 59.454	724.118 132.290	903.226 128.155	——	——	——
Blood	Mean 1 SD	0.007 0.004	0.008 0.008	0.016 0.008	0.020 0.017	0.017 0.013	0.018 0.011	0.010 0.000	0.019 0.005	0.010 0.004
Heart	Mean 1 SD	0.028 0.012	0.046 0.013	0.079 0.011	0.167 0.015	0.213 0.006	0.128 0.024	0.062 0.015	0.018 0.011	0.027 0.012
Liver	Mean 1 SD	0.020 0.010	0.035 0.082	0.179 0.115	0.656 0.136	1.125 0.038	0.738 0.100	0.430 0.037	0.369 0.041	0.165 0.016
Spleen	Mean 1 SD	0.031 0.030	0.069 0.028	0.369 0.121	0.556 0.078	0.913 0.221	0.538 0.061	0.321 0.087	0.469 0.011	0.253 0.112
Lung	Mean 1 SD	0.013 0.000	0.017 0.018	0.206 0.020	0.115 0.027	0.093 0.012	0.068 0.015	0.094 0.013	0.071 0.021	0.062 0.010
Kidney	Mean 1 SD	0.033 0.030	0.057 0.028	0.137 0.021	0.074 0.027	0.109 0.017	0.065 0.013	0.039 0.007	0.039 0.034	0.021 0.010
Stomach	Mean 1 SD	0.009 0.004	0.017 0.003	0.404 0.011	0.030 0.008	0.039 0.017	0.038 0.011	0.026 0.015	0.049 0.021	0.036 0.019
Intestine	Mean 1 SD	0.023 0.010	0.099 0.038	0.154 0.035	0.159 0.063	0.292 0.127	0.225 0.051	0.113 0.031	0.176 0.063	0.148 0.030
Pancreas	Mean 1 SD	0.008 0.000	0.024 0.002	0.125 0.011	0.312 0.015	0.057 0.024	0.051 0.01	0.043 0.021	0.035 0.012	0.055 0.022
Brain	Mean 1 SD	0.017 0.009	0.006 0.011	0.028 0.01	0.099 0.023	0.065 0.024	0.033 0.011	0.037 0.012	0.041 0.021	0.024 0.014
Thyroid	Mean 1 SD	0.016 0.000	0.034 0.007	0.026 0.011	0.044 0.013	0.032 0.015	0.042 0.021	0.032 0.011	0.041 0.022	0.035 0.013
Testis	Mean 1 SD	0.022 0.010	0.021 0.013	0.038 0.022	0.021 0.008	0.042 0.010	0.058 0.031	0.029 0.008	0.033 0.016	0.036 0.011
Bone	Mean 1 SD	0. 233 0.021	0.377 0.035	0.369 0.121	1.049 0.236	1.518 0.049	1.081 0.211	0.930 0.084	0.569 0.308	0.656 0.182
Muscle	Mean 1 SD	0.011 0.000	0.035 0.010	0.034 0.015	0.043 0.017	0.052 0.023	0.041 0.026	0.034 0.017	0.029 0.021	0.025 0.020

ID: injected dose. 0.5, 1, 2, 4, 8, 16, 32, 48, and 64 d were time at which animals were anesthetized and sacrificed, blood was collected, tissues were weighed, and radioactivity was quantified. *n* = 5 mice at each time point.

**Table 2:** Biodistribution of ^32^P-CP particle in nude mice bearing U87MG glioma after intratumor injection of ^32^P-CP colloids (*n* = 5).

Tissue	Radioactivity distribution (%ID/g)									
		0.5 d	1 d	2 d	4 d	8 d	16 d	32 d	48 d	64 d
Tumor	Mean 1 SD	429.1 110.2	455.60 265.72	570.5 159.4	470.76 218.05	525.57 52.21	676.25 61.15	——	——	——
Blood	Mean 1 SD	0.057 0.021	0.073 0.035	0.079 0.010	0.046 0.011	0.022 0.047	0.048 0.014	0.02 0.011	0.079 0.014	0.070 0.012
Heart	Mean 1 SD	0.171 0.052	0.124 0.013	0.232 0.151	0.207 0.105	0.148 0.049	0.097 0.054	0.009 0.035	0.246 0.113	0.183 0.071
Liver	Mean 1 SD	0.253 0.110	0.146 1.077	1.690 0.745	1.656 1.076	2.918 0.076	1.219 0.171	1.122 2.037	2.629 0.041	2.465 0.016
Spleen	Mean 1 SD	0.324 0.197	0.249 0.128	0.559 0.121	1.241 0.052	0.913 0.021	0.769 0.061	0.334 0.087	0.469 0.011	0.356 0.019
Lung	Mean 1 SD	0.233 0.103	0.163 0.018	0.239 0.078	0.256 0.127	0.433 0.212	0.512 0.015	0.222 0.013	0.041 0.021	0.052 0.010
Kidney	Mean 1 SD	0.192 0.074	0.153 0.028	0.269 0.121	0.256 0.127	0.019 0.017	0.071 0.092	0.210 0.007	0.269 0.034	0.056 0.019
Stomach	Mean 1 SD	0.056 0.034	0.097 0.003	0.016 0.011	0.033 0.008	0.019 0.017	0.113 0.011	0.241 0.015	0.049 0.021	0.036 0.019
Intestine	Mean 1 SD	0.203 0.109	0.038 0.012	0.154 0.035	0.159 0.063	0.156 0.127	0.136 0.051	0.408 0.031	0.176 0.063	0.248 0.030
Pancreas	Mean 1 SD	0.030 0.024	0.101 0.002	0.049 0.011	0.074 0.015	0.077 0.024	0.182 0.01	0.020 0.021	0.035 0.012	0.055 0.022
Brain	Mean 1 SD	0.017 0.019	0.026 0.021	0.011 0.010	0.036 0.023	0.024 0.014	0.013 0.011	0.008 0.012	0.013 0.021	0.024 0.014
Thyroid	Mean 1 SD	0.016 0.021	0.077 0.007	0.026 0.011	0.044 0.013	0.026 0.015	0.022 0.021	0.012 0.011	0.021 0.022	0.035 0.013
Testis	Mean 1 SD	0.022 0.012	0.041 0.013	0.038 0.022	0.021 0.008	0.042 0.010	0.018 0.031	0.029 0.008	0.033 0.016	0.036 0.011
Bone	Mean 1 SD	0.762 0.421	2.343 0.035	2.369 0.121	3.856 0.236	4.004 1.093	3.541 0.211	2.605 0.084	2.869 0.308	2.656 0.182
Muscle	Mean 1 SD	0.007 0.003	0.083 0.010	0.034 0.015	0.043 0.017	0.046 0.023	0.092 0.026	0.053 0.017	0.049 0.021	0.045 0.020

ID: injected dose. 0.5, 1, 2, 4, 8, 16, 32, 48, and 64 d were time at which animals were anesthetized and sacrificed, blood was collected, tissues were weighed, and radioactivity was quantified. *n* = 5 mice at each time point.

## Abbreviations

^32^P: Phosphorus-32
^68^Ga: Gallium-68
^18^F: Fluorine-18
CT: Computed tomography
PET: Positron emission tomography
SPECT: Single photon emission computed tomography
^32^P-CP: ^32^P-chromic phosphate
^32^P-CP-PLGA: ^32^P-chromic phosphate-polylactide-co-glycolide
%ID/g: Percent injected dose per gram
FDG: Fludeoxyglucose.
